# Oral Health Among Swedish Patients with Substance Use Disorders – A Comparative, Cross-Sectional Study

**DOI:** 10.3290/j.ohpd.a44032

**Published:** 2020-07-04

**Authors:** Sonja Rafat, Mesfin Tessma, Björn Klinge, Stefan Borg, Patricia De Palma

**Affiliations:** a PhD Student, Department of Dental Medicine, Karolinska Institutet, Huddinge, Sweden. Implemented and conceived the study and in charge of overall direction and planning; wrote the manuscript with input from all authors.; b Department of Learning, Informatics, Management and Ethics (LIME), Karolinska Institutet, Solna, Sweden; Faculty of Odontology, Malmö University, Malmö, Sweden. Performed the calculations, analysis, wrote part of the results, and gave feedback in writing the manuscript.; c Department of Dental Medicine, Karolinska Institutet, Huddinge, Sweden; Faculty of Odontology, Malmö University, Malmö, Sweden. Gave feedback in writing manuscript and analysing the data.; d Department of Neurosciences Karolinska Institutet, Solna, Sweden. Gave feedback in writing manuscript and analysing the data.; e Department of Dental Medicine, Karolinska Institutet, Huddinge, Sweden. Designed the model and the computational framework; conceived the study and in charge of overall direction and planning.

**Keywords:** cannabis, DMFT, heroin, periodontal health, substance use disorder

## Abstract

**Purpose:**

This study explored the oral health of individuals with substance use disorders and examined the relationship between oral health and type and number of years of substance use disorder.

**Materials and Methods:**

This comparative cross-sectional study comprised patients with one of four groups of substance use disorders – alcohol, cannabis, central nervous system stimulants (CNSS), and opiates. All participants underwent a dental examination and were included in the study based on their clinical findings.

**Results:**

Of 95 participants, 79 (83%) were male and 37 (39%) were homeless. Statistically significant difference between the groups was observed in 6–12-mm periodontal pocket depths (p <0.05), as were differences in oral mucosal changes (p <0.001). Statistically significantly lower proportions were observed in the cannabis group for Mob G:0 and Mob G:1 and Furcation G:1 compared to the CNSS and opiate groups; the proportion of Furcation G:0 was significantly lower in the alcohol group compared to the cannabis group. Analysis of variance (ANOVA) revealed statistically significant between-group differences in age, number of years of substance use disorder, number of teeth, and decayed, missing and filled teeth (DMFT). When controlling for age and gender, substance type was found to be a statistically significant predictor of number of teeth (B = –4.4; 95% CI: –8.1 to -0.38; p = 0.03) and DMFT (B = 2.1; 95% CI: 0.86 to 3.3; p = 0.001).

**Conclusions:**

These results indicate poor oral health among individuals with substance use disorders. It seems that oral health problems are lower among abusers of cannabis than of CNSS, alcohol and opiates.

Substance use disorders and oral disease are two conditions that exacerbate socioeconomic problems.^19,30^ Among the adult population (between the ages 15 and 64), substance use disorders have an estimated global prevalence of 3.4–6.6%; cannabis is the most common illicit substance use disorder, both globally (2.6–5.0%) and in Sweden (2.5%).^24,30^ Oral diseases affect more than 3.9 billion people globally. This statistic includes periodontitis, severe tooth loss and untreated caries lesions.^21,25,33^ Although international studies have shown that patients with substance use disorders are more likely to suffer from oral disease than the general population,^4,15,23^ to our knowledge, these conditions have not been well studied, especially in Sweden. Other factors, such as homelessness and smoking, have been reported to have a negative effect on oral health.^5,6,17^

This article is the first in a series of studies focusing on substance use disorders in Stockholm County with special reference to oral health.

The hypothesis of this study was that there is a negative relationship between oral health and type and the number of years of substance use. The aim was to describe and compare clinical findings of oral health and its relationship between type and number of years of individuals with substance use disorders in Stockholm County.

## MATERIALS AND METHODS

### Study Population

The study population comprised individuals with substance use disorders seeking care between January 2012 and December 2015 at Addiction Treatment Centers (ATCs, managed by the Stockholm Center for Dependency Disorders) in Stockholm County.

**Fig 1:**
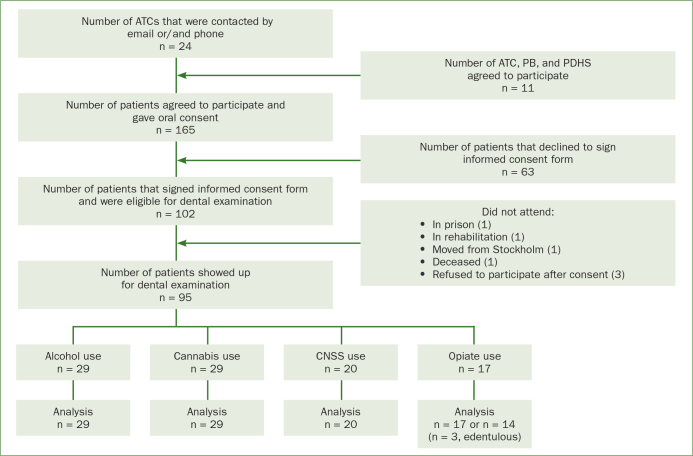
Flow chart of participation in the clinical examination. ATCs = Addiction Treatment Centers; PB = Pelarbacken; PDHS = Public Dental Health Service; CNSS = central nervous system stimulants (amphetamine, cocaine and khat).

Ninety-five participants agreed to participate and signed an informed consent form before the dental examination (Fig 1). The participants were informed that the study was optional and that they could cancel their participation at any time. Inclusion criteria were (1) seeking treatment at an ATC in Stockholm County, (2) age between 18 and 65 years, and (3) fluent in Swedish or English. The exclusion criterion was any severe psychiatric disorder (eg, major depressive, bipolar, or schizoaffective disorder). The Regional Ethics Review Board in Stockholm approved the study (Dnr [Daybook number]: 2010/1806-31/4).

### Data Collection

Various ATCs in Stockholm County were contacted by e-mail and/or phone to arrange an information visit with Sonja Rafat (SR), one of the authors. Information targeted the staff, to inform them about the study and about encouraging subjects to participate. The participants were recruited directly from the ATC while SR was visiting, or they contacted SR by e-mail or telephone to book a dental appointment. Participants completed these questionnaires: the Addiction Severity Index (ASI), the Alcohol Use Disorders Identification Test (AUDIT), the Drug Use Disorders Identification Test (DUDIT), Oral Health Habits, and the Global Assessment of Function scale (GaF scale). These questionnaires will be presented in another study. Participants also underwent a clinical dental examination at one of the ATCs while SR was visiting, at Pelarbacken (PB, which is part of Ersta diakoni, an organisation providing health and dental services for homeless people), or at the Public Dental Health Service in Stockholm (PDHS). PB was chosen as an examination place due to its central location in Stockholm Municipality; participants from the various ATCs in the county would be able to easily reach PB. SR examined all 95 participants, 5 at an ATC, 82 at PB, and 8 at the PDHS. Patients with different clinical diagnosis and at different stages of treatment were included in the study based on their clinical findings. After the examination, PB provided free dental care for the homeless participants, while participants who were not homeless but were in need of dental treatment were referred to a dental clinic of their choice.

### Clinical Dental Examination

The clinical examination (described next) required approximately 1.5 h for each patient, and findings were recorded on an examination protocol. When possible, clinical photographs were taken, depending on whether sufficient time remained, and the participant was willing. A few participants declined clinical photography due to embarrassment over their teeth or because they did not want any photos taken. The radiographic examination included four bitewing radiographs or radiographs adapted to the dentition of the participant (findings will be reported in a future study). All participants were encouraged to visit PB for a full examination that included a radiographic examination.

Calibration between SR, the clinical examiner, and Patricia De Palma (PDP), the experienced author, was done before the start of the study to examine inter-rate reliability. Cohen’s Kappa was used to assess the inter-rater reliability of the two raters. The interrater reliability for the raters was found to be Kappa = 0.9, indicating a very high agreement. The outcome of the calibration was a consensus in the treatment of the patient group and an understanding of the patient’s state of mind and lifestyle.

### Dentition

Based on the number of teeth present (excluding third molars),^5^ the participants were classified in one of three groups: good dental status, 28–21 teeth; poor dental status, 20–1 teeth; and edentulous, 0 teeth. The number of root remnants were also recorded.

### Periodontal Examination

The periodontal examination comprised measurements of (1) probing pocket depth (PPD) with a periodontal probe scaled in mm (LM Dental AB; 23–52b Si Perio, 2-mm scale); (2) bleeding on probing (BoP): if bleeding occurred within 10–15 s of probing, a positive score was recorded; (3) visible plaque (as the plaque index (PI), recorded after drying the teeth with air according to the methods of Ainamo and Bay^1^); (4) presence of calculus on each tooth; (5) tooth mobility (Mob), using a finger and an instrument handle; and (6) furcation involvements (Furcation) according to the criteria of Lindhe and Nyman.^18^ PPD, BoP and PI were recorded on the mesial, buccal, distal and lingual sites of each tooth. Mob and Furcation were recorded for each mobile tooth in grade (G): 1, 2 and 3. Root remnants and dental implants were excluded from all measurements.

### Community periodontal index

The community periodontal index (CPI)^32^ is a screening tool that has been frequently used in describing the epidemiology of the periodontal condition. We divided the mouth into sextants, as defined by these tooth numbers: 17–14, 13–23, 24–27, 37–34, 33–43 and 44–47; and recorded the CPI score for each sextant.

### Caries and restoration

All tooth surfaces were examined clinically and radiographically for caries according to the criteria of the World Health Organization^31^ and Palmer and Knutsson.16 Decayed, missing and filled teeth (DMFT) and presence of restorations (dentures, crowns/bridges, and dental implants) were also recorded.

### Oral mucosa

Oral mucosa and soft tissues were scored according to World Health Organization criteria^31^ for white anomalies (hyperkeratosis, leukoplakia), red anomalies (hyperplasia, ulceration, erythema) and snuff lesions.

### Type of Substance Use Disorder

All participants were seeking treatment and/or rehabilitation for a specific substance that they abused and were assigned to one of four groups: alcohol, cannabis, central nervous system stimulants (CNSS; amphetamine, cocaine and khat), or opiates (heroin). Information on the type of substance use disorder was also extracted from the ASI questionnaire when needed.

### Number of years of substance use disorder

Number of years of substance use disorder was extracted from the ASI.

### Statistical Analysis

Descriptive statistics are presented as means, standard deviations, and medians (ranges) for numerical variables, or as frequencies or percentages for categorical variables. The between-group differences in subject characteristics were examined using a chi-square test or Fischer’s exact test for categorical variables and the one-way analysis of variance (ANOVA) for continuous variables. Levine’s test checked the assumption of equality of variance for the ANOVA. If the overall ANOVA was statistically significant, further analysis using a Tukey HSD or Bonferroni or Games-Howell post-hoc pairwise test for comparisons was done. Games-Howell was used if there was violation of the equality of variance assumption. For ordinal variables, the non-parametric Kruskal–Wallis test was used. To examine trend in proportions in mobility, furcation, BoP and PI, we performed the chi-squared test. For evaluating effect size, the difference between means, 95% confidence intervals, partial eta squared (η^2^), and R^2^ were used. Multiple linear regression controlled for potential confounders. Residual plots, normal probability plots and Cook’s distance assessed model assumptions. A Cook’s distance (D) >1 provided a strong indication of outlier problems, and D >4/n, where n was the sample size, indicated a possible problem. A variation inflation factor (VIF) greater than 4 was the cut-off criterion for deciding when a given independent variable displayed too great of a multicollinearity problem. All statistical analyses were done using the Statistical Package for the Social Sciences (SPSS) for Windows (version 23; IBM, NY, USA) with the level of statistical significance set at 0.05.

### Sample size

We estimated the sample size needed to detect a minimum mean DMFT difference of 4 among the groups using a 5% statistical significance level and 80% power.^5,28^ Assuming a non-response rate of 25%, a minimum sample size of 92 subjects was required for the study.

## RESULTS

### Main Findings

ANOVA revealed statistically significant differences between groups in age, number of years of substance use disorder, number of teeth and DMFT. Multiple linear regression analyses showed that substance type was a statistically significant predictor of number of current teeth (B = –4.4, 95% CI: –8.1 to –0.38, p = 0.03) and DMFT (B = 2.1, 95% CI: 0.86 to 3.3, p = 0.001) when controlled for age and gender.

### Participant Characteristics

In total, 165 subjects agreed to participate and gave their oral consent; 102 subjects signed an informed consent form (Fig 1). Of the 102 participants, 95 participants (79 males and 16 females) showed up for the dental examination. Depending on what substance the participants were in treatment for at ATC, they were included in one of the four groups: alcohol (n = 29), cannabis (n = 29), CNSS (amphetamine, cocaine, khat [n = 20]), and opiates (heroin, [n = 17]). The mean age in the study population was 40.3 years, and 39% were homeless. Table 1 presents participant characteristics of the study groups. The proportion of males was greater in the cannabis and alcohol groups than in the CNSS and opiate groups (p = 0.052). Of the four groups, the cannabis group had the lowest mean age, the highest proportion of males, and the lowest proportion of homeless individuals (Table 1). The proportion of homeless individuals varied significantly between groups (p <0.001), with a lower proportion of homeless individuals in the cannabis group than in the CNSS, opiate or alcohol groups. Pairwise comparisons using a Bonferroni correction revealed statistically significant differences between the cannabis group and the CNSS (p <0.001) and opiate groups (p <0.001) where the cannabis group has a lower proportion of homeless. We observed no statistically significant between-group differences in the proportions of individuals who reported smoking or taking snuff.

**Table 1:** Participant characteristics of the study groups

Variable	Alcohol use (n = 29)	Cannabis use (n = 29)	CNSS use (n = 20)	Opiate use (n = 17)
**Mean (SD)**				
Age, years***	46.9 (10.0)	29.8 (7.4)	43.8 (12.0)	42.8 (11.2)
No. of years***	18.7 (11.4)	11.6 (8.8)	21.2 (13.1)	13.6 (8.5)
No. of teeth	23.2 (6.7)	26.8 (2.5)	19.9 (8.5)	16.9 (9.9)
**Number (%)**				
Gender, male	24 (83)	28 (97)	14 (70)	13 (76)
Homeless, yes***	11 (38)	3 (10)	13 (65)	10 (59)
Smoking, yes	20 (69)	16 (55)	13 (65)	13 (77)
Taking snuff, yes	15 (52)	14 (48)	5 (25)	4 (24)

*** p <0.001; No. of years = number of years of substance use disorder, No. of teeth = number of teeth.

### Periodontal Examination

Table 2 presents the results of the periodontal examination by group and participants. A significant difference was observed in 6-12-mm PPD [χ^2^ = 10.5, df = 3, p = 0.014]. Post hoc analysis revealed that there was statistically significantly higher proportion of 6–12 mm PPD in the CNSS group (p = 0.003) and opiate groups (p = 0.006) compared to the cannabis group. We did not observe statistically significant differences between the alcohol and cannabis (p = 0.40) and the CNSS and opiate groups (p = 0.93). Kruskal–Wallis analysis revealed that there was a statistically significant difference between groups in the proportion of Mob G:0, Mob G: 1 and Mob G2. Post hoc pairwise analysis revealed that the cannabis group was significantly different from the CNSS (p <0.001) and opiate groups (p = 0.03) in Mob 0 and Mob G:1. The proportion of Mob G:0 was higher in the cannabis group but lower for Mob G:1 compared to the other groups. Similar statistically significant differences were observed between the cannabis and opiate groups in Furcation G:0 (p = 0.03) and cannabis and alcohol groups in Furction G:1 (p = 0.04) (Table 2). The proportion of Furcation G:0 was higher in the cannabis group but lower for Furcation G:1 compared to the other groups. We did not observe statistically significant differences in BoP and PI. PPD, Mob, and Furcation were recorded as a positive finding when a site had a depth of 4–5 mm or 6–12 mm: Mob G:1, G:2, or G:3; or Furcation G:1, G:2, or G:3. Chi-squared test for trend revealed the odds of mobility increased with increased degree for mobility in cannabis (Mantel–Haenszel chi-square for linear trend = 4.9, p = 0.03) and CNSS groups (Mantel–Haenszel chi-square for linear trend = 6.0, p = 0.01) and for furcation in the cannabis group (Mantel-Haenszel Chi-square for linear trend = 6.7 p = 0.009). We did not observe significant linear trend in BoP and PI.

**Table 2:** **Table 2** Results of the periodontal examination, number and percentage of participants by variable and substance type group

Variable	Alcohol use^Ϯ^(n = 29)	Cannabis^Ϯ^ use^Ϯ^(n = 29)	CNSS use^Ϯ^(n = 20)	Opiate use^Ϯ^(n = 14)
**Number (%)** ^a^				
PPD				
4–5 mm	29 (100)	29 (100)	20 (100)	14 (100)
6–12 mm*	11 (38)	8 (28)	14 (70)	10 (71)
Mob, G				
0***	13 (45)	21 (72)	2 (10)	5 (36)
1***	16 (55)	8 (28)	18 (90)	12 (86)
2*	5 (17)	2 (7)	8 (40)	4 (29)
3	2 (7)	0 (0)	4 (20)	2 (14)
Furcation, G				
0*	9 (31)	19 (66)	8(40)	4 (29)
1*	19 (66)	10 (35)	11(55)	13 (93)
2*	3 (10)	2 (7)	7 (35)	4 (29)
3	1 (3)	0 (0)	1 (5)	0 (0)
BoP, yes				
<20	10 (34)	15 (52)	8 (40)	5 (36)
20–50	8 (28)	5 (17)	4 (20)	3 (21)
>50	11 (38)	9 (31)	8 (40)	6 (43)
PI, yes				
<20	2(7)	0 (0)	0 (0)	0 (0)
20–50	4 (14)	11 (38)	5 (25)	2 (12)
>50	23 (79)	18 (62)	15 (75)	12 (71)
Calculus	28 (97)	28 (97)	20 (100)	14 (100)
CPI 0	0 (0)	0 (0)	0 (0)	0 (0)
CPI 1	0 (0)	0 (0)	0 (0)	0 (0)
CPI 2	0 0	0 (0)	0 (0)	0 (0)
CPI 3	18 (62)	21 (72)	6 (30)	4 (29)
CPI 4	11 (37.9)	8(27.6)	14 (70)	10 (71.4)

* p <0.05; *** p <0.001; Ϯ The number in the table may exceed the total number of participants because a patient may have more than one event; a Percentages are for each subcategory of a variable of each drug type.

PPD = probing pocket depth; BoP = bleeding on probing; PI = visible plaque index; Mob, G = tooth mobility grade; Furcation, G = furcation involvements grade;

CPI = community periodontal index; CPI 0 = healthy; CPI 1 = BoP; CPI 2 = calculus; CPI 3 = PPD 4 or 5 mm; CPI 4 = PPD, >6 mm.

Because three individuals in the opiate group were edentulous, the opiate group was recalculated to 14 participants for the periodontal analyses (Fig 1).

We observed no statistically significant between-group differences in CPI 0, CPI 1 or CPI 2. However, there were statistically significant differences between the groups in CPI 3 and CPI 4 (Table 2).

### Dentition, DMFT, Restoration and Oral Mucosa

Table 3 presents data on dentition, DMFT, restorations, bruxism and oral mucosa anomalies. The opiate group had a higher percentage (47%, n = 8) of participants in the edentulous and poor dental status groups. Members of the opiate group also had a higher mean DMFT (20.7) and more restorations (57%, n = 8) than members of the other groups. The cannabis group had no participants with dentures; 97% (n = 28) of its participants placed in the good dental status group.

**Table 3:** Results from the clinical examination and oral mucosa inspection by substance type group; numbers and percentages of study participants unless otherwise indicated

Variable	Alcohol use(n= 29)	Cannabis use(n = 29)	CNSS use(n = 20)	Opiate use(n = 17)
**Clinical examination**				
**Dentition**				
Root remnants	8 (28)	3 (10)	5 (25)	4 (24)
Edentulous	0 (0)	0 (0)	0 (0)	3 (18)
Poor dentition	4 (14)	1 (3)	7 (35)	5 (29)
Good dentition	25 (86)	28 (97)	13 (65)	9 (53)
DMFT***				
**mean (SD)**	14.1 (7.2)	9.9 (6.4)	15.5 (6.9)	20.7 (7.9)
**Restoration**				
Dentures	1 (3)	0 (0)	3 (15)	2/14 (14)
Crowns/bridges	9 (31)	6 (21)	2 (10)	4/14 (29)
Dental implants	0 (0)	1 (3)	0 (0)	2/14 (14)
Bruxism	23 (79)	27 (93)	17 (85)	13/14 (93)
**Oral mucosa**				
White anomalies	13 (45)	5 (17)	11 (55)	10 (59)
Red anomalies	2 (7)	1 (3)	4 (20)	1 (6)
Snuff lesion	14 (48)	14 (48)	5 (25)	5 (29)
No anomalies	0 (0)	9 (31)	0 (0)	1 (6)

*** p <0.001; DMFT = decayed, missing, and filled teeth; * Percentages may not add up to 100 due to rounding error.

Fischer’s exact test revealed a statistically significant difference in oral mucosal changes (p <0.001) between the cannabis and the other substance types (alcohol, CNSS and opiate groups). Pairwise comparisons using a Bonferroni correction also showed statistically significant differences between the cannabis group and the other groups: cannabis vs alcohol, p = 0.001; cannabis vs CNSS, p = 0.004; cannabis vs opiate, p <0.001, in all comparisons we observed lower proportion in the cannabis group.

As in measurements of periodontal status, the opiate group was recalculated to include only 14 participants. The findings of DMFT and the restorations are presented per participant. In DMFT measurements, root remnants were classified as missing teeth.

### Age, number of teeth, DMFT and number of years of substance use disorder

ANOVA revealed statistically significant between-group differences in age, number of years of substance use disorder, number of teeth and DMFT (Table 4). The one-way ANOVA revealed statistically significant differences in ages between the groups (F(3, 91) = 16, p <0.001, partial ^2^ = 0.34). Post-hoc comparisons using the Tukey honestly significant difference (HSD) test found mean age in the cannabis group to be significantly lower than in the alcohol (mean difference = –17.1, 95% CI: –23.2 to –10.9, p <0.001), CNSS (mean difference = –14.0, 95% CI: –22.3 to –5.8, p <0.001), or opiate (mean difference = –13.0, 95% CI: –21.3 to –4.6; p <0.001) groups.

**Table 4:** **Table 4** Results of the one-way ANOVA for the dependent variables of age; number of years of substance use disorder; number of teeth; and decayed, missing, and filled teeth (DMFT)

Source of variation	Sum of squares	df	Mean square	F	p value	Partialη2
**Age**						
Between	4798	3	1599	15.9	<0.001	0.34
Within	9139	91	100			
**No. of years**						
Between	1413	3	471	4.2	0.008	0.12
Within	10 229	91	112			
**No. of teeth**						
Between	1226	3	409	8.5	<0.001	0.22
Within	4378	91	48			
**DMFT**						
Between	1108	3	369	7.5	<0.001	0.20
Within	4455	91	49			

df = degree of freedom; Partial η2 = partial eta squared; No. of years = no. of years of substance use disorder; No. of teeth = number of teeth.

Number of years of substance use disorder differed significantly between the different substance type groups ([F(3, 91) = 4.2, p = 0.008, partial η^2^ = 0.12]; Table 4). Post-hoc comparisons using the Tukey HSD test revealed that mean number of years of substance use disorder in the cannabis group was significantly lower than in the CNSS group (mean difference = –9.6, 95% CI: –17.7 to –1.5, p = 0.01), indicating greater number of years of substance use disorder for the CNSS group. However, the differences between the cannabis and alcohol groups (mean difference = –7.1, 95% CI: –14.4 to –0.83, p = 0.06) and the cannabis and opiate groups (mean difference = –2.0, 95% CI: –10.5 to 6.5, p = 0.93) were non-statistically significant.

Statistically significant differences were also detected in number of teeth (F[3, 91] = 8.5, p < 0.001, partial η^2^ = 0.22; Table 4). Post-hoc comparisons with the Tukey HSD test revealed that the mean number of teeth in the cannabis group was significantly higher than in the CNSS (mean difference = 6.9, 95% CI: 1.5 to 12.4, p = 0.009) and the opiate (mean difference = 9.9, 95% CI: 3.0 to 16.9, p = 0.004) groups. The difference between the cannabis and alcohol groups, however, was lower (mean difference = 3.6, 95% CI: –004.4 to 7.2; p = 0.05). The results indicated that the cannabis group had a higher number of teeth than the CNSS and opiate groups.

Statistically significant differences were observed in DMFT (F[3,91] = 8.0, p <0.001, partial η^2^ = 0.20; Table 4). Post-hoc comparisons using the Tukey HSD test revealed significantly lower levels of DMFT in the cannabis group compared with the CNSS (mean difference = –5.6, 95% CI = –10.9 to –0.28; p <0.001) and opiate (mean difference = –10.8, 95% CI = –15.5.3 to –4.3; p = 0.03) groups, but not when compared with the alcohol group (mean difference = –4.2, 95% CI: –9.0 to 0.60, p = 0.11). The results indicated that the cannabis group had a lower mean DMFT score than the CNSS and opiate groups.

Regression analyses were done to control for potential confounders. When controlled for age and gender, the analyses revealed that substance type was a statistically significant predictor of two variables: number of teeth (B = –2.5, 95% CI –3.7 to –1.2, p = 0.01) and DMFT (B = 2.1, 95% CI = 0.86 to 3.3; p = 0.001; Table 5).

**Table 5:** Results of the multiple linear regression for the dependent variables number of teeth and decayed, missing, and filled teeth (DMFT)

Variable	B	SE	t	p value	95% CI	R-squared
**No. of teeth**						0.33
Age	–0.29	0.05	–5.8	<0.001	–0.40, –0.19	
Gender	–0.84	1.8	–0.47	0.60	–4.4, 2.7	
Group	–2.5	0.62	–4.0	<0.001	–3.7, –1.2	
**DMFT**						0.32
Age	3.1	0.05	5.6	<0.001	2.0, 0.42	
Gender	0.29	1.8	0.16	0.87	–3.3, 3.8	
Group	2.1	0.62	3.4	0.001	0.86, 3.3	

B = coefficient (B); SE = standard error; t = t test; CI = confidence interval. Group: 1 = Alcohol, 2 = Cannabis, 3 = CNSS, 4 = Opiate.

## DISCUSSION

This cross-sectional study describes and compares the oral health of a group of individuals with substance use disorders living in Stockholm County, Sweden. Age, number of years of substance abuse, and oral health findings differed significantly between the substance use disorder groups alcohol, cannabis, CNSS and opiates. When age and gender were controlled, substance use disorder type group was also a significant predictor of number of teeth and DMFT.

Most studies on substance use disorders have an older study population,^5,23^ but the age profile in the present study is in line with another study.^15^ The gender distribution in our sample with females representing only 17% of the study population is also in line with similar studies.^4,5,23^

This study is in contrast with the Jönköping study (2015),^22^ which indicated that, in the general Swedish population, the older age groups would retain increasingly more of their teeth over time. However, our study is in line with the De Palma et al study (2005),^5^ which found that, compared with the general population, number of teeth among substance use disorders tend to decrease at a more rapid rate.

Prevalence of periodontitis is estimated to be 25–40% in the general Swedish population, and of the most severe form of periodontitis, 5–15% globally.^8,9,12^ Substance use disorders have reported to have a negative effects on the periodontium, however, the effects have not been well studied. A study done in New Zealand^29^ examined the effects of cannabis smoking on periodontal health and concluded that cannabis could be an independent risk factor for periodontal disease. Several studies have reported that patients with heroin and methamphetamine use disorders are more likely to have poor oral and periodontal health compared with patients with other substance use disorders.^7,15,20^ The periodontal status of this study population is in line with previous studies,^7,11,13,15,20^ which reported that patients who abuse heavier substance such as amphetamine, cocaine, heroin and khat have a greater risk of developing periodontitis than those who abuse alcohol or cannabis. Patients with substance use disorders are also more likely to have periodontal problems compared to the general Swedish population.^9,12,22^

Untreated caries, which is also an oral disease condition, is the most prevalent non-communicable disease and affects 35% of the adult population globally; in Western Europe, the prevalence is 35.8%.^14^

Studies conducted in the UK^4^ and Ireland^23^ compared the DMFT scores between alcohol only and alcohol and drug abuser groups and reported lower DMFT scores for the alcohol only group.^4^ Other studies done in the US^27^ and China^7^ have reported a high DMFT score among individuals with substance use disorders. The present study also showed that individuals with a substance use disorder not only have more severe periodontal disease and fewer teeth with age, but also have a higher DMFT score compared to the general Swedish population.^9,22^ The results also indicated that oral health status differed depending on the type and number of years a specific substance was abused. The participants in the cannabis group had abused for a shorter period of time, were the youngest in age, and had more teeth and a healthier periodontal status compared to the other groups. BoP and PI scores in the cannabis group, however, were higher than in the other groups.

A large portion of the participants were homeless (39%), which was unsurprising in light of the findings of previous research by two of the authors.^5^ Despite a homeless lifestyle, a large portion of the study population also smoked tobacco and abused snuff; these factors, besides the substance use disorder, could also contribute to poor oral health.^5,6,17^

The clinical implications of the study may be pointed out as follows. Increasing the availability of oral health services and introducing regular follow-up is of paramount importance to prevent serious complications in all groups. The cannabis group has lower risk and are younger, thus earlier intervention and creating awareness may benefit the group; the CNSS and opiate groups are at higher risk. Therefore it is recommended that additional attention is paid to these untreated dental caries high-risk groups with the most urgent need for oral healthcare.

This study, however, has several limitations. It would have been beneficial to record periodontitis as done in other studies.^13,15,22,29^ We chose CPI as a periodontal screening tool due to the absence of data on clinical attachment level (CAL) and of a radiographic examination, which would have been necessary for diagnosing moderate and severe periodontitis. Presenting both clinical and radiological assessments is important to support the findings of this study. However, a radiographic examination was not possible for all participants due to their dental status or willingness to cooperate.

This is an additional limitation of the study and we recommend that future studies employ both clinical and radiological assessments in their work. Another limitation of the survey is lack of control group from the general population. In addition, important predictors such as education, social status or employment status were not included in this study. These variables are relevant determinates that may affect the level of oral health for these kinds of patients. We recommend that future studies include control group from the general population and relevant determinants of oral health.

The most common oral symptom when abusing any drug is xerostomia^2,3,10,26^ which can be easily examined by dental staff. However, measuring saliva flow was not done in this study but is recommended in future studies. The aim of this study was to describe and compare oral health in a sample of substance use disorders in Stockholm County and to examine the relationship between oral health and type and number of years of substance use disorder. The hypothesis was that there would be a negative relationship between oral health and types and number of years of substance use disorder. The results suggest that there are associations between oral health and type and number of years of substance use disorder in many of the variables.

## CONCLUSION

This study found poor oral health among patients with substance use disorders, especially for those in the CNSS and opiate groups. The results suggest lower levels of oral disease among cannabis abusers compared with CNSS, alcohol and opiate abusers; however, the lower mean age and the lower number of years of substance use disorders in the cannabis group compared with the other groups may be a factor to consider in relation to the low level of disease. In future studies, more detailed periodontal measurements, such as CAL, to diagnose more validated periodontal conditions would be preferable.

## References

[ref1] Ainamo J, Bay I (1975). Problems and proposals for recording gingivitis and plaque. Int Dent J.

[ref2] Antoniazzi RP, Sari AR, Casarin M, Moraes CMB, Feldens CA (2017). Association between crack cocaine use and reduced salivary flow. Braz Oral Res.

[ref3] Cho CM, Hirsch R, Johnstone S (2005). General and oral health implications of cannabis use. Aust Dent J.

[ref4] Dasanayake AP, Warnakulasuriya S, Harris CK, Cooper DJ, Peters TJ, Gelbier S (2010). Tooth decay in alcohol abusers compared to alcohol and drug abusers. Int J Dent.

[ref5] De Palma P, Frithiof L, Persson L, Klinge B, Halldin J, Beijer U (2005). Oral health of homeless adults in Stockholm, Sweden. Acta Odontol Scand.

[ref6] de Pereira M, Oliveira L, Lunet N (2014). Caries and oral health related behaviours among homeless adults from Porto, Portugal. Oral Health Prev Dent.

[ref7] Du M, Bedi R, Guo L, Champion J, Fan M, Holt R (2001). Oral health status of heroin users in a rehabilitation centre in Hubei province, China. Community Dent Health.

[ref8] Dye BA (2012). Global periodontal disease epidemiology. Periodontol 2000.

[ref9] Edman K, Ohrn K, Holmlund A, Nordstrom B, Hedin M, Hellberg D (2012). Comparison of oral status in an adult population 35–75 year of age in the county of Dalarna, Sweden in 1983 and 2008. Swed Dent J.

[ref10] Friedlander AH, Marder SR, Pisegna JR, Yagiela JA (2003). Alcohol abuse and dependence: psychopathology, medical management and dental implications. J Am Dent Assoc.

[ref11] Hach M, Holm-Pedersen P, Adegboye AR, Avlund K (2015). The effect of alcohol consumption on periodontitis in older Danes. Int J Dent Hyg.

[ref12] Hugoson A, Sjodin B, Norderyd O (2008). Trends over 30 years, 1973–2003, in the prevalence and severity of periodontal disease. J Clin Periodontol.

[ref13] Jamieson LM, Gunthorpe W, Cairney SJ, Sayers SM, Roberts-Thomson KF, Slade GD (2010). Substance use and periodontal disease among Australian Aboriginal young adults. Addiction.

[ref14] Kassebaum NJ, Bernabe E, Dahiya M, Bhandari B, Murray CJ, Marcenes W (2015). Global burden of untreated caries: a systematic review and metaregression. J Dent Res.

[ref15] Kayal RA, Elias WY, Alharthi KJ, Demyati AK, Mandurah JM (2014). Illicit drug abuse affects periodontal health status. Saudi Med J.

[ref16] Klein H, Panlmer CE, Knutsson JW (1938). Studies on dental caries. 1. Dental status and dental needs of elementary school. Public Health Rep.

[ref17] Kulkarni V, Uttamani JR, Bhatavadekar NB (2016). Comparison of clinical periodontal status among habitual smokeless-tobacco users and cigarette smokers. Int Dent J.

[ref18] Lindhe J, Nyman S (1975). The effect of plaque control and surgical pocket elimination on the establishment and maintenance of periodontal health. A longitudinal study of periodontal therapy in cases of advanced disease. J Clin Periodontol.

[ref19] Listl S, Galloway J, Mossey PA, Marcenes W (2015). Global economic impact of dental diseases. J Dent Res.

[ref20] Ma H, Shi XC, Hu DY, Li X (2012). The poor oral health status of former heroin users treated with methadone in a Chinese city. Med Sci Monit.

[ref21] Marcenes W, Kassebaum NJ, Bernabe E, Flaxman A, Naghavi M, Lopez A (2013). Global burden of oral conditions in 1990–2010: a systematic analysis. J Dent Res.

[ref22] Norderyd O, Koch G, Papias A, Kohler AA, Helkimo AN, Brahm CO (2015). Oral health of individuals aged 3–80 years in Jonkoping, Sweden during 40 years (1973–2013). II. Review of clinical and radiographic findings. Swed Dent J.

[ref23] O’Sullivan EM (2012). Dental health of Irish alcohol/drug abuse treatment centre residents. Community Dent Health.

[ref24] Remstedt M, Sundin E, Landberg J, Raninen J, STAD-Report 55. Stockholm: Stockholm for the prevention of alcohol and drug abuse (STAD) (2014).

[ref25] Richards D (2013). Oral diseases affect some 3.9 billion people. Evid Based Dent.

[ref26] Rommel N, Rohleder NH, Koerdt S, Wagenpfeil S, Hartel-Petri R, Wolff KD (2016). Sympathomimetic effects of chronic methamphetamine abuse on oral health: a cross-sectional study. BMC Oral Health.

[ref27] Shetty V, Mooney LJ, Zigler CM, Belin TR, Murphy D, Rawson R (2010). The relationship between methamphetamine use and increased dental disease. J Am Dent Assoc.

[ref28] Silverstein SJ (1973). Relation between social drug use-abuse and dental disease in California, U.S.A. Community Dent Oral Epidemiol.

[ref29] Thomson WM, Poulton R, Broadbent JM, Moffitt TE, Caspi A, Beck JD (2008). Cannabis smoking and periodontal disease among young adults. JAMA.

[ref30] UNDC. World drug report 2012 (2012). United Nations.

[ref31] WHO, World Health Organization (2013). Oral Health Surveys: Basic Methods.

[ref32] WHO, World Health Organization (1997). Oral health surveys: basic methods.

[ref33] Vos T, Barber RM, Bell B, Bertozzi-Villa A, Biryukov S, Bolliger I (2015). Global, regional, and national incidence, prevalence, and years lived with disability for 301 acute and chronic diseases and injuries in 188 countries, 1990–2013: a systematic analysis for the Global Burden of Disease Study 2013. Lancet.

